# Impact of Nutritional Status on Outcomes of Stroke Survivors: A Post Hoc Analysis of the NHANES

**DOI:** 10.3390/nu15020294

**Published:** 2023-01-06

**Authors:** Hsueh-Yi Lu, Ue-Cheung Ho, Lu-Ting Kuo

**Affiliations:** 1 Department of Industrial Engineering and Management, National Yunlin University of Science and Technology, Yunlin 640, Taiwan; 2 Division of Neurosurgery, Department of Surgery, National Taiwan University Hospital Yunlin Branch, Yunlin 640, Taiwan; 3 Division of Neurosurgery, Department of Surgery, National Taiwan University Hospital, Taipei 100, Taiwan

**Keywords:** stroke, nutrition, mortality, survival

## Abstract

Stroke, a neurological emergency, is a leading cause of death and disability in adults worldwide. In acute or rehabilitative stages, stroke survivors sustain variable neurological recovery with long-term disabilities. The influence of post-stroke nutritional status on long-term survival has not been confirmed. Using the United States National Health and Nutrition Examination Survey data (2001–2010), we conducted a matched-cohort analysis (929 and 1858 participants in stroke and non-stroke groups, respectively) to investigate the influence of nutritional elements on post-stroke survival. With significantly lower nutrient consumption, the mortality risk was 2.2 times higher in stroke patients compared to non-stroke patients (Kaplan–Meier method with Cox proportional hazards model: adjusted hazard ratio, 2.208; 95% confidence interval: 1.887–2.583; *p* < 0.001). For several nutritional elements, the lower consumption group had significantly shorter survival than the higher consumption stroke subgroup; moreover, stroke patients with the highest 25% nutritional intake for each nutritional element, except moisture and total fat, had significantly shorter survival than non-stroke patients with the lowest 25% nutrition. Malnutrition is highly prevalent in stroke patients and is associated with high mortality rates. The dynamic change in energy requirements throughout the disease course necessitates dietary adjustment to ensure adequate nutritional intake.

## 1. Introduction

Stroke is a neurological emergency that necessitates immediate medical management or surgical intervention and is attributed to acute brain injury that may be caused by two major vascular insults: blood vessel occlusion or rupture. Although substantial progress in the development of therapeutic strategies has been made in the past few years, stroke remains one of the leading causes of death and disability in adults worldwide [1]. Patients who survive their first stroke experience have varied degrees of neurological recovery during the acute and rehabilitative stages [2,3,4,5,6]; however, approximately 34–54% of survivors will have long-term moderate-to-severe disabilities [7,8,9,10]. In the rehabilitative stage, a multidisciplinary team approach, which includes nutritional support, can be beneficial for functional outcomes [11,12,13]. Stroke is prevalent in older patients, who usually have other medical problems, and are prone to malnutrition [14]. However, the detailed benefits of post-stroke nutritional status on long-term survival have not been conclusively determined, especially in light of variations arising from the different tools used for nutritional assessment and the timing of post-stroke assessments [15]. This study was performed to evaluate the difference in nutritional status between patients with and without stroke, and to explore the influence of various nutritional elements on survival in patients with a stroke.

## 2. Materials and Methods

### 2.1. Data Sources

The data analyzed in this study were obtained from the National Health and Nutrition Examination Survey (NHANES) 2001–2010 database, which comprises information recorded from a series of cross-sectional, stratified, multistage probability surveys of the civilian, non-institutionalized US population. The NHANES was conducted by the National Center for Health Statistics (NCHS) within the United States Centers for Disease Control and Prevention (CDC) and aimed to evaluate the health and nutritional status of the US population. The study survey, a continuous program wherein every 2 years represent one cycle, was administered through face-to-face interviews and physical examinations that were conducted in a mobile examination center (MEC). Further information about the NHANES is available at https://www.cdc.gov/nchs/nhanes/index.htm (accessed on 3 September 2022) [16].

### 2.2. Participants

From the 52,198 participants of the continuous NHANES surveys from 2001 to 2010, we preliminarily selected 1094 participants who reported that a doctor or other health professional had informed them that they had a stroke. The date designated as the index date to investigate the risk of mortality was estimated from the questionnaire of “How long has the participant had stroke problems (number of days)? We excluded participants who were <18 years or had missing values; thus, 929 participants with stroke (stroke group) were eligible for inclusion in the analysis. By using 2:1 age (±3 years)- and sex-based matching, the controls (patients without stroke) were randomly selected from the same dataset to include participants who did not have a stroke.

### 2.3. Nutritional Status

Dietary intake from foods was estimated through a single 24 h dietary recall interview [17], and information on dietary supplement intake, such as vitamins, was obtained from supplement questionnaires that were administered as part of the NHANES household interview. The consumption frequency, duration, and dosage were recorded for each supplement that was used in the past 30 days [16,18]. The average daily intake of nutrients from dietary supplements was calculated on the basis of the supplement consumption frequency and dosage.

### 2.4. Mortality Data

NHANES mortality files were linked to the National Death Index by using a probabilistic matching algorithm to determine the mortality status. Follow-up of the participants continued until death, and participants for whom a death record was not matched were assigned “alive” status during the follow-up period [19].

### 2.5. Statistical Analysis

Demographic and nutritional variables for patients in the stroke or non-stroke groups were expressed as the frequency (percentage) or mean (±standard deviation (SD)), with the chi-squared or Student’s *t*-test performed for comparison. Demographic characteristics included sex, age, and body mass index (BMI). Continuous variables were examined for normality of distribution using the Kolmogorov D test. Cumulative incidence curves for mortality were plotted using the Kaplan–Meier method, and the differences in the curves of the stroke and non-stroke groups were tested using a log-rank test. The Cox proportional hazards model was used to measure the main effect of nutrition in stroke patients at the time to death. Hazard ratios (HRs) and 95% confidence intervals (CIs) were estimated using Cox regression. Variables with significant values in the univariate model were further examined using a Cox regression model. Patients with stroke were further divided into quartile intervals based on nutritional consumption, and the adjusted HRs were estimated by comparing the interquartile range (IQR, midpoint 50%) with the lowest to the first quartile (Q1-L, first quarter) and with the third quartile to the highest quartile (Q3-H, fourth quarter). All statistical tests were two-sided, and a *p*-value of 0.05 was considered significant. The power based on the sample size with the required 5% significance level was >99%. Statistical analyses were performed using the SPSS 26 for Windows (SPSS, Inc., Chicago, IL, USA).

### 2.6. Ethics Statement

This study was conducted in accordance with the NHANES research ethics protocols that were approved by the NCHS Research Ethics Review Board. All the participants provided informed consent. The detailed study design and ethics statement were approved by the institutional review board for public use, and the data files can be found on the NHANES website [20].

## 3. Results

### 3.1. Participant Characteristics

The mean age of the stroke group was 67.46 ± 13.79 years, and males comprised 50.16% (Table 1). Similarly, the non-stroke group comprised 1858 participants (mean age 66.63 ± 13.84 years, and 50.10% male). The distributions of baseline covariates (age and sex) were similar in the stroke and non-stroke groups. In the stroke and non-stroke groups, 266 (28.63%) and 407 (21.90%) participants, respectively, died. Patients in the stroke group had significantly lower nutrient consumption, including those of calorie, protein, carbohydrate, total fat, total monounsaturated fatty acids (MFA), total polyunsaturated fatty acids (PFA), vitamin E, vitamin B1, vitamin B2, niacin, vitamin B6, vitamin C, vitamin K, phosphorus, magnesium, iron, zinc, copper, potassium, and selenium. The absolute levels of each nutritional element in each group of patients (Q1-L, IQR, and Q3-H) are presented in Table 2. The daily calorie intake in patients with stroke were <1146 in Q1-L group, 1146–2058 in IQR group, and >2058 kcal in Q3-H group. The daily sodium consumption in patients with stroke <1786 in Q1-L group, 1786–3391 in IQR group, and >3391 mg in Q3-H group.

### 3.2. Comparison of Survival between the Stroke and Non-Stroke Participants

On analysis using the Kaplan–Meier method with a Cox proportional hazards model, the adjusted HR (aHR 2.208; 95% CI: 1.887–2.583; *p* < 0.001) indicated that the mortality risk was 2.2 times higher in stroke patients compared to non-stroke patients (Table 3). The aHR of 2.208 was used as the baseline ratio for further investigations to examine the mortality risk between the stroke and non-stroke groups for different consumption variables, which were stratified into three quartile intervals (Q1-Lowest (Q1-L), IQR, Q3-Highest (Q3-H)). Mortality decreased as the dietary consumption of most nutrients increased, and this trend was observed in both the stroke and non-stroke groups. Furthermore, with increased consumption of some nutritional elements, the intergroup differences in survival between the stroke and non-stroke groups increased (Table 3). For example, with regard to magnesium consumption, participants in the Q3-H quartile had a relatively higher OR (aHR 3.347) among the stroke and non-stroke groups as compared with the baseline group (aHR 2.208); thus, non-stroke patients may benefit from increased magnesium consumption than stroke patients. Moreover, these differences were identified for the increased consumption of calories (aHR 2.711), sodium (aHR 3.240), and moisture (aHR 2.930).

### 3.3. Dietary Consumption on Survival among Stroke Patients

**Figure 1:**
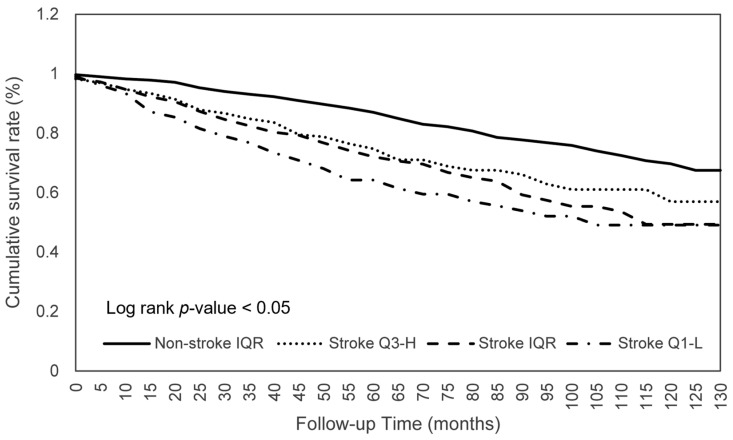
Calorie consumption in relation to the cumulative mortality rates. Abbreviations: Q1-L, Q1-lowest; IQR, interquartile range; Q3-H, Q3-highest.

**Figure 2:**
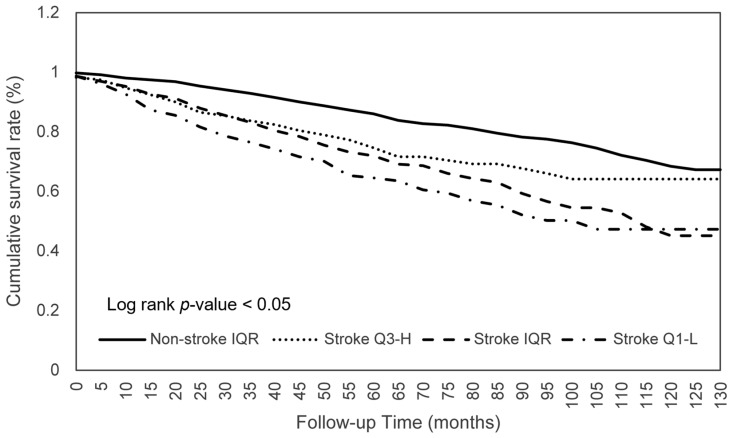
Protein consumption in relation to the cumulative mortality rates. Abbreviations: Q1-L, Q1-lowest; IQR, interquartile range; Q3-H, Q3-highest.

**Figure 3:**
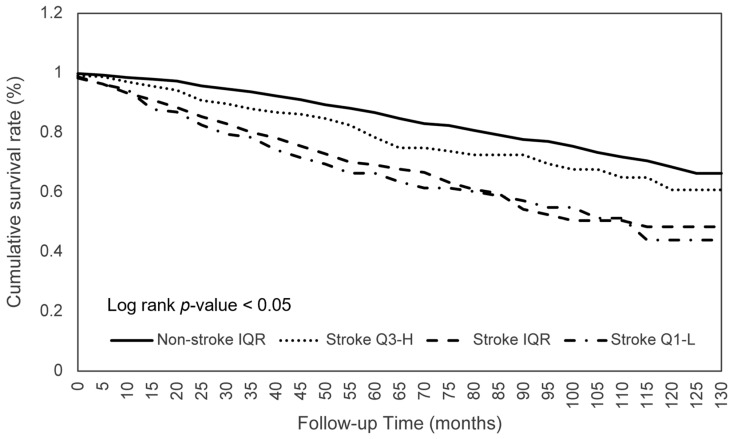
Total fat consumption in relation to the cumulative mortality rates. Abbreviations: Q1-L, Q1-lowest; IQR, interquartile range; Q3-H, Q3-highest.

**Figure 4:**
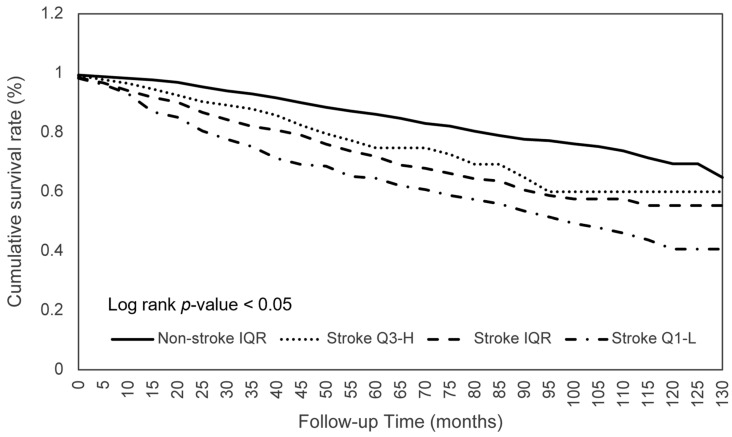
Moisture consumption in relation to the cumulative mortality rates. Abbreviations: Q1-L, Q1-lowest; IQR, interquartile range; Q3-H, Q3-highest.

The effects of consumption on the survival of patients with stroke were further investigated. As shown in Table 4, stroke patients within the Q1-L of calorie consumption had a significantly worse survival (aHR 0.744; *p* < 0.05) than those within the IQR, which indicated that the mortality risk of participants who consumed the middle 50% of calories was 0.744 times lower than those with lower 25% calorie consumption (Figure 1). Similar significant ORs were found for protein (aHR 0.681; *p* < 0.01), phosphorus (aHR 0.714; *p* < 0.05), selenium (aHR 0.703; *p* < 0.05), and caffeine (aHR 0.740; *p* < 0.05) consumption (Figure 2). Another significant finding was that stroke patients with an IQR of fat consumption had a significantly lower survival (aHR 0.713; *p* < 0.05) than those within the Q3-H, and this trend was observed in the comparison of the survival in the Q3-H and Q1-L groups with regard to protein consumption (Figure 3). For other nutritional elements, the survival between the patients in the Q3-H and IQR groups and in the Q3-H and Q1-L groups did not differ significantly (Figure 4).

### 3.4. Dietary Consumption on Survival between Stroke Q3-H and Non-Stroke Q1-L Patients

There was a higher mortality risk in the stroke group compared with that in the non-stroke group in each quartile; accordingly, we specifically examined the effect of dietary consumption on survival between stroke Q3-H and non-stroke Q1-L participants to determine if a significant difference existed (Table 5). Except for the intake of moisture and total fat, stroke patients with the highest 25% nutritional intake for each of the nutritional elements had significantly lower mortality rates than non-stroke patients with the lowest 25% nutritional intake.

## 4. Discussion

We examined the difference in nutritional status between stroke and non-stroke groups and demonstrated that stroke patients had less nutrient intake than non-stroke patients. Additionally, the overall mortality rate decreased as nutrient consumption increased for most nutrients, and this trend was observed in both the stroke and non-stroke groups.

Nutrition is an important issue in patients with stroke, regardless of the clinical stage. Furthermore, nutrition has been proposed as one of the modifiable risk factors for stroke prevention [21,22,23,24,25,26] and is viewed as an important determinant of post-stroke neurological recovery [27,28,29,30,31,32]. Malnutrition is highly prevalent and is often unrecognized in stroke patients, with a reported prevalence of 6.1–79% [33,34,35,36,37,38]. Differences in stroke type, disease severity, nutrition screening tools, and assessment timing could have contributed to this wide prevalence range. In alignment with the results of previous research [35,39,40], we demonstrated that, when compared to non-stroke patients, stroke patients were malnourished and had less nutrient intake, including calories, carbohydrates, proteins, fat, vitamins, electrolytes, and microelements. This phenomenon was maintained even when the nutrient intake was stratified into three quartile intervals (Q1-L, IQR, Q3-H). The negative impact of malnutrition on the neurological recovery and survival of stroke patients has been increasingly recognized, despite conflicting results [14,30,41,42,43,44,45,46,47,48,49]. This highlights the importance of early identification of patients with stroke who are malnourished or at risk of malnutrition to provide timely nutritional intervention.

Current guidelines recommend that enteral feeding should be administered within 7 days of acute stroke, and that nutritional supplements should be considered for patients who are undernourished or at risk of malnutrition [50]. However, the guidelines do not specify the tools that should be utilized to screen for malnutrition or the nutritional supplements that should be provided. Various screening tools for malnutrition have been developed in past decades for use in different populations and clinical settings [51,52,53], of which only a few, including the Malnutrition Universal Screening Tool (MUST) [43,54], Controlling Nutritional Status Score (CONUT) [46,48], Geriatric Nutritional Risk Index (GNRI) [48], Prognostic Nutritional Index score (PNI) (48), and Nutritional Risk Screening 2002 (NRS-2002) [46], have been validated in stroke populations and are accepted for predicting clinical outcomes in stroke patients. Each screening tool was calculated from different nutritional parameters, in consideration of different disease status, and was divided into different categories based on the severity of malnutrition. The wide variation in the prevalence of malnutrition that has been determined by various screening tools leads to different thresholds for nutritional intervention, which makes it difficult to interpret the results appropriately. Thus, there is a need for the development of valid and reliable nutrition screening tools that are specifically for use in stroke patients.

Notably, regardless of the screening tools that were used, an increasing number of stroke patients were identified as having malnutrition or worsening nutritional status during hospitalization despite the detection of a malnourished status at admission [34,35,38,55]. This is counterintuitive to our understanding that early detection allows for an early intervention for better clinical outcomes or prevention of malnutrition. Different energy requirements are required throughout the course of the disease, and these depend on the patient’s stroke type, post-stroke complications, and ability to participate in daily activities. Furthermore, alterations in the brain metabolism caused by different disease status or pharmacological interventions complicate the nutritional assessment of stroke patients. In recent decades, a few studies have reported conflicting results for the association between stroke type and resting energy expenditure (REE), wherein the REE was reduced in ischemic stroke [56,57], or that the REE was not altered significantly throughout the disease course [58,59,60,61,62]. These results should be interpreted cautiously because of the different study time frames and therapeutic interventions, such as sedation or hypothermia, which might have led to a hypometabolic state. In contrast to studies on ischemic stroke, the REE levels increased in patients with hemorrhagic stroke [62,63,64], especially aneurysmal subarachnoid hemorrhage (aSAH) [62,63,65,66,67,68,69]. This phenomenon could be explained by an increase in metabolism, which is attributed to the effect of post-injury inflammation. In the hypermetabolic state, increased energy requirements worsen the mismatch between food consumption and energy expenditure, and thereby lead to negative energy balance and poor clinical outcomes. In our study, the overall mortality rate was higher in the stroke group than that in the non-stroke group. Within each group, patients with higher nutrient consumption had a lower mortality rate, and this trend existed across all types of nutrients. The HR in each quartile of nutrient consumption varied with regard to the overall HR, and this could be explained by the varying degrees of the effect of nutrient consumption on the reduction of the mortality risk. Nonetheless, the positive effect of nutrient consumption on mortality should not be neglected, and all types of nutrients should be consumed adequately; this is especially important in stroke patients. Moreover, although the amount of nutrient consumption in stroke Q3-H was not much higher than that in non-stroke Q1-L, we demonstrated that the mortality rate of the stroke Q3-H was lower than that of the non-stroke Q1-L with regard to most nutrients. This observation highlights the fact that energy balance is determined by both nutrient consumption and energy requirements. An increased REE due to different types of strokes, in combination with an inability to meet the energy requirements, will lead to a negative energy balance. Therefore, it is important to recognize the dynamic changes in energy requirements throughout the disease course and to adjust the dietary program accordingly.

Indirect calorimetry (IC) is a noninvasive and quantitative tool for the clinical measurement of energy expenditure; it is considered the gold standard for REE assessment and is widely used in clinical research. The Harris–Benedict equation (HBE) is proposed to estimate an individual’s REE if a device for IC is unavailable. Many studies have compared HBE with IC in different clinical settings, including stroke, albeit with equivocal outcomes [61,64,70,71,72]. Two studies investigated the effects of stroke characteristics, including stroke size, type, location, and severity, on the REE, and although the REE did not vary with stroke characteristics, the authors advocated further confirmation with larger subgroups [49,58]. Together with the aforementioned factors that affect the REE, it is possible to develop a tool or equation that considers factors such as stroke type, imaging characteristics, patient profile, time period, or even intervention to accurately predict a patient’s REE. For this aim to be actualized, it is important to consider all aspects when estimating the patient’s energy requirement and to continuously review the patient’s nutritional status for preventing malnutrition.

In addition to identifying changes in energy requirements in different stroke populations and stages, it is important to identify patients who are at risk of malnutrition. Many risk factors are associated with malnutrition in stroke patients, including dysphagia, consciousness disturbance, motor deficit, visuospatial impairment, depression, malnutrition on admission, and pharmacotherapy [73,74,75]. Early identification of patients at risk of malnutrition by screening for the aforementioned risk factors could enable clinicians to undertake timely, appropriate nutritional management for better clinical outcomes.

Some studies have reported the positive effect of dietary supplements on clinical outcomes [12,13,76,77,78,79,80]; however, few studies have investigated patients’ actual nutrient intake and the association with functional outcomes and mortality. Among the nutrients, proteins are one of the most important elements that improve the post-stroke neurological recovery [76,77], and this is especially true of certain high-quality amino acids [81,82,83,84]. Owing to their hypermetabolic state, which leads to decreased muscle strength and even physical function, stroke patients should receive adequate protein supplementation. In agreement with previous reports, we found that the protein intake significantly affected mortality among stroke patients. Thus, protein should be considered as of paramount importance when planning nutritional intervention in stroke patients to meet their daily energy requirements. Furthermore, vitamins play a role in neurological recovery owing to the antioxidative effect in the acute period, wherein increased oxidative stress suppresses protein synthesis and thus impairs brain recovery [27,85,86,87]. Vitamin B supplementation in stroke patients mitigates oxidative stress and reduces the risk of post-stroke depression [78,88]. In addition, supplementation of vitamins C, D, and E induces positive effects on antioxidant activity, neuroprotection, and functional recovery [89,90,91,92]. Moreover, some studies have evaluated other nutrients and mineral supplements, such as potassium/magnesium [80], zinc [93], and omega-3 polyunsaturated fatty acids [79], in stroke patients and have reported promising clinical outcomes. In our study, we found a positive effect of different kinds of electrolytes and mineral supplements on clinical outcomes, although this trend did not reach statistical significance. Notably, our study provided a large-scale overview of nutritional profiles in stroke patients, and this could facilitate the development of appropriate, individualized nutritional plans.

Ethnic differences in REE [94,95,96], stroke prevalence [97,98] and stroke outcome [99] had been reported. This factor is also raised as an important issue due to biological variability and different socioeconomic status when the nutrition literature is being conducted [100,101]. Understanding the differences among stroke patients with various ethnic backgrounds is also important to guide the development of a nutrition screening tool and nutritional intervention. More studies are needed to elucidate the ethnic issues in accessing nutrition status of stroke patients.

Our study had several limitations. First, the data were acquired from the NHANES study, which mainly comprised a civilian noninstitutionalized US population. Therefore, the generalizability of the results to regions outside the US is questionable.

Second, some patients with stroke have cognitive impairments or are severely disabled and live in hospitals or institutions. These institutionalized populations were excluded from the survey, which may have biased the interpretation of the nutritional status in patients with stroke. Third, nutritional status was acquired using a self-reported questionnaire, which raises the concern of recall bias. Finally, this study comprised a series of surveys and was a repeated cross-sectional study. The causality between malnutrition for each dietary element and clinical outcomes, including mortality and neurological status, requires further investigation in a large-scale prospective cohort study.

## 5. Conclusions

Malnutrition is highly prevalent in stroke patients and is associated with higher mortality rates. Proteins are one of the most important elements that improve post-stroke neurological recovery and should be considered when planning nutritional intervention in stroke patient. The dynamic change in energy requirements throughout the disease course necessitates the adjustment of dietary programs and highlights the need for adequate dietary intake. The nutritional profiles should be further researched to develop appropriate individualized nutritional plans.

## Figures and Tables

**Table 1:** Demographic and dietary consumption characteristics in participants with and without stroke.

Variables	Stroke *n* = 929	Non-Stroke *n* = 1858	*p*-Value
Age, years (mean ± SD)	67.46 ± 13.79	66.63 ± 13.84	0.135
Sex, male (%)	466 (50.16)	931 (50.10)	0.123
Body mass index	27.73 ± 2.46	28.45 ± 5.52	**<0.001**
Calories (kcal)	1701.71 ± 802.15	1860.41 ± 825.6	**<0.001**
Protein (g)	65.39 ± 34.42	72.17 ± 36.65	**<0.001**
Carbohydrate (g)	210.88 ± 101.93	229.57 ± 107.07	**<0.001**
Total fat (g)	65.42 ± 39.15	70.54 ± 39.11	**0.001**
Total SFA (g)	21.66 ± 14.06	22.39 ± 13.49	0.183
Total MFA (g)	24.06 ± 15.35	25.82 ± 15.56	**0.005**
Total PFA (g)	13.73 ± 9.71	15.21 ± 10.3	**<0.001**
Cholesterol (g)	260.62 ± 215.13	268.52 ± 216.51	0.363
Vitamin E (mg)	5.86 ± 4.34	6.55 ± 4.94	**<0.001**
Retinol (μg)	414.73 ± 679.61	413.76 ± 750.08	0.974
Vitamin A, RAE (μg)	574.82 ± 728.45	622.73 ± 835.68	0.137
Vitamin B1 (mg)	1.34 ± 0.73	1.46 ± 0.84	**<0.001**
Vitamin B2 (mg)	1.83 ± 0.94	1.98 ± 1.04	**<0.001**
Niacin (mg)	19.09 ± 10.52	20.84 ± 11.85	**<0.001**
Vitamin B6 (mg)	1.58 ± 0.96	1.76 ± 1.03	**<0.001**
Vitamin B12 (μg)	4.49 ± 7.03	5.09 ± 8.49	0.061
Vitamin C (mg)	77.44 ± 83.51	91.15 ± 91.83	**<0.001**
Vitamin K (μg)	84.37 ± 142.6	99.1 ± 176.51	**0.027**
Calcium (mg)	739.34 ± 478.52	766.16 ± 470.14	0.158
Phosphorus (mg)	1066.8 ± 528.15	1165.91 ± 548.79	**<0.001**
Magnesium (mg)	234.9 ± 115.1	265.62 ± 136.01	**<0.001**
Iron (mg)	13.01 ± 7.44	14.37 ± 8.39	**<0.001**
Zinc (mg)	9.74 ± 6.34	10.9 ± 10.25	**0.002**
Copper (mg)	1.11 ± 1.2	1.26 ± 1.4	**0.004**
Sodium (mg)	2788 ± 1535	2883 ± 1482	0.116
Potassium (mg)	2286 ± 1086	2555 ± 1195	**<0.001**
Selenium (μg)	86.62 ± 49.08	95.33 ± 54.41	**<0.001**
Caffeine (mg)	148.15 ± 194.25	150.44 ± 186.83	0.763
Theobromine (mg)	35.09 ± 75.5	30.16 ± 66.79	0.079
Moisture (g)	2155 ± 1299	2168 ±1197	0.785
Death	266 (28.63)	407 (21.90)	**<0.001**
Follow-up, months	54.40 ± 33.26	82.24 ± 30.89	**<0.001**

Abbreviations: SD, standard deviation; SFA, saturated fatty acids; MFA, monounsaturated fatty acids; PFA, polyunsaturated fatty acids; RAE, retinol activity equivalent.

**Table 2:** Quartiles of dietary consumption with and without stroke.

	Stroke (*n* = 929)	Non–Stroke (*n* = 1858)	Total (*n* = 2787)
Calories (kcal)			
Q1-L	262–1146	164–1297	164–1249
IQR	1146–2058	1297–2297	1249–2237
Q3-H	2058–7301	2297–6798	2237–7301
Protein (g)			
Q1-L	0.63–42.48	7.51–48.02	0.63–46.32
IQR	42.48–79.54	48.02–89.10	46.32–86.67
Q3-H	79.54–308.16	89.10–399.74	86.67–399.74
Total fat (g)			
Q1-L	0.00–38.9	2.47–42.81	0.00–41.36
IQR	38.9–82.04	42.81–90.26	41.36–87.36
Q3-H	82.04–406.09	90.26–322.20	87.36–406.09
Total SFA (g)			
Q1-L	0.00–12.14	0.41–12.80	0.00–12.64
IQR	12.14–27.54	12.80–28.72	12.64–28.47
Q3-H	27.54–117.70	28.72–105.76	28.47–117.70
Total MFA (g)			
Q1-L	0.00–13.87	0.45–14.90	0.00–14.47
IQR	13.87–30.62	14.90–33.18	14.47–32.32
Q3-H	30.62–161.31	33.18–153.77	32.32–161.31
Total PFA (g)			
Q1-L	0.00–7.31	0.77–7.89	0.00–7.72
IQR	7.31–17.70	7.89–19.48	7.72–18.86
Q3-H	17.70–110.97	19.48–96.90	18.86–110.97
Vitamin E (mg)			
Q1-L	0.00–3.00	0.23–3.43	0.00–3.26
IQR	3.00–7.37	3.43–8.39	3.26–8.10
Q3-H	7.37–38.96	8.39–55.76	8.10–55.76
Phosphorus (mg)			
Q1-L	0–704	77–797	0–758
IQR	704–1352	797–1442	758–1416
Q3-H	1352–4636	1442–4523	1416–4636
Magnesium (mg)			
Q1-L	15–150	19–173	15–166
IQR	150–294	173–330	166–317
Q3-H	294–773	330–1451	317–1451
Sodium (mg)			
Q1-L	41–1786	122–1851	41–1820
IQR	1786–3391	1851–3665	1820–3565
Q3-H	3391–12861	3665–11862	3565–12861
Selenium (μg)			
Q1-L	0.00–54.20	4.8–60.4	0.00–58.10
IQR	54.20–109.00	60.4–116.9	58.10–114.50
Q3-H	109.00–422.10	116.9–651.7	114.50–651.70
Caffeine (mg)			
Q1-L	0–10	0–10	0–10
IQR	10–208	10–213	10–213
Q3-H	208–2389	213–1971	213–2389

Abbreviations: Q1-L, Lowest-Q1; Q3-H, Q3-Highest; IQR, interquartile range; SFA, saturated fatty acids; MFA, monounsaturated fatty acids; PFA, polyunsaturated fatty acids.

**Table 3:** Impact of dietary consumption on survival with and without stroke.

	Stroke (*n* = 929)	Non-Stroke (*n* = 1858)	Adjusted HR (95% CI)
Mortality (%)	266 (28.6) ^a^	407 (21.1)	2.208 (1.887–2.583)
Mortality with nutritional consumption (%)			
Calories (kcal)			
Q1-L	80 (34.5)	133 (28.7)	2.075 (1.564–2.753)
IQR	129 (27.7)	203 (21.9)	2.135 (1.706–2.672)
Q3-H	57 (24.6)	71 (15.3)	2.711 (1.905–3.857)
Protein (g)			
Q1-L	80 (34.5)	122 (26.3)	2.316 (1.736–3.089)
IQR	132 (28.4)	207 (22.3)	2.156 (1.727–2.692)
Q3-H	54 (23.3)	78 (16.8)	2.292 (1.614–3.256)
Total fat (g)			
Q1-L	76 (32.5)	121 (26.1)	2.172 (1.617–2.917)
IQR	141 (30.5)	208 (22.4)	2.398 (1.930–2.981)
Q3-H	49 (21.0)	78 (16.8)	1.968 (1.372–2.822)
Total SFA (g)			
Q1-L	66 (28.4)	120 (25.9)	1.909 (1.405–2.595)
IQR	146 (31.7)	216 (23.3)	2.299 (1.858–2.854)
Q3-H	54 (22.9)	71 (15.3)	2.479 (1.732–3.548)
Total MFA (g)			
Q1-L	74 (32.3)	123 (26.5)	2.181 (1.620–2.936)
IQR	141 (30.1)	205 (22.1)	2.357 (1.896–2.930)
Q3-H	51 (22.0)	79 (17.0)	2.049 (1.436–2.924)
Total PFA (g)			
Q1-L	75 (32.3)	114 (24.6)	2.161 (1.604–2.912)
IQR	133 (28.6)	216 (23.3)	2.204 (1.769–2.746)
Q3-H	58 (25.0)	77 (16.6)	2.381 (1.688–3.359)
Vitamin E (mg)			
Q1-L	67 (28.6)	119 (25.6)	1.923 (1.415–2.613)
IQR	147 (31.7)	221 (23.8)	2.279 (1.844–2.817)
Q3-H	52 (22.4)	67 (14.4)	2.561 (1.776–3.694)
Phosphorus (mg)			
Q1-L	82 (35.2)	120 (25.8)	2.439 (1.825–3.258)
IQR	127 (27.4)	207 (22.3)	2.031 (1.624–2.540)
Q3-H	57 (24.6)	80 (17.2)	2.436 (1.727–3.437)
Magnesium (mg)			
Q1-L	76 (32.6)	125 (26.9)	1.934 (1–447–2.585)
IQR	126 (27.2)	218 (23.5)	2.036 (1.628–2.545)
Q3-H	64 (27.6)	64 (13.8)	3.347 (2.357–4.754)
Sodium (mg)			
Q1-L	80 (34.5)	110 (23.7)	2.348 (1.752–3.148)
IQR	121 (26.0)	233 (25.1)	1.850 (1.480–2.313)
Q3-H	65 (28.0)	64 (13.8)	3.240 (2.285–4.593)
Selenium (μg)			
Q1-L	80 (34.5)	125 (26.9)	2.128 (1.599–2.831)
IQR	126 (27.1)	199 (21.4)	2.174 (1.732–2.728)
Q3-H	60 (25.9)	83 (17.9)	2.455 (1.754–3.437)
Caffeine (mg)			
Q1-L	81 (34.9)	114 (24.3)	2.492 (1.864–3.333)
IQR	128 (27.5)	212 (23.2)	1.951 (1.562–2.435)
Q3-H	57 (24.6)	81 (17.1)	2.537 (1.795–3.584)
Moisture (g)			
Q1-L	99 (42.5)	145 (31.3)	2.059 (1.591–2.666)
IQR	125 (26.9)	203 (21.9)	2.053 (1.637–2.575)
Q3-H	42 (18.1)	59 (12.7)	2.930 (1.949–4.405)

^a^ Number (%) of deaths. *p* values for all adjusted HRs are <0.001. Abbreviations: Q1-L, Lowest-Q1; Q3-H, Q3-Highest; IQR, interquartile range; SFA, saturated fatty acids; MFA, monounsaturated fatty acids; PFA, polyunsaturated fatty acids; HR, hazard ratio; CI, confidence interval.

**Table 4:** Impact of dietary consumption on survival of stroke patients.

Stroke	Log Rank	Adjusted HR (95% CI)		
(*n* = 929)	*p*-Value	Q1-L to IQR	IQR to Q3-H	Q1-L to Q3-H
Calories	0.043	0.744 * (0.559–0.990)	1.062 (0.771–1.464)	0.866 (0.719–1.043)
Protein	0.026	0.681 ** (0.511–0.907)	1.081 (0.778–1.501)	0.816 * (0.673–0.991)
Total fat	0.002	0.940 (0.711–1.244)	0.713 * (0.512–0.993)	0.837 (0.694–1.010)
Total SFA	0.073	1.101 (0.822–1.475)	0.807 (0.588–1.109)	0.926 (0.767–1.118)
Total MFA	0.008	0.887 (0.668–1.177)	0.774 (0.558–1.074)	0.841 (0.697–1.016)
Total PFA	0.214	0.832 (0.625–1.107)	0.881 (0.646–1.202)	0.863 (0.742–1.029)
Vitamin E	0.101	0.992 (0.740–1.328)	0.780 (0.568–1.071)	0.877 (0.729–1.056)
Phosphorus	0.036	0.714 * (0.537–0.949)	1.113 (0.810–1.529)	0.851 (0.711–1.019)
Magnesium	0.767	0.773 (0.579–1.033)	1.104 (0.813–1.500)	0.870 (0.729–1.037)
Sodium	0.177	0.787 (0.593–1.045)	1.142 (0.840–1.554)	0.995 (0.837–1.181)
Selenium	0.143	0.703 * (0.527–0.937)	1.164 (0.850–1.593)	0.866 (0.721–1.040)
Caffeine	0.009	0.740 * (0.560–0.978)	1.013 (0.736–1.392)	0.848 (0.713–1.009)
Moisture	0.004	0.783 (0.597–1.027)	1.203 (0.838–1.726)	0.942 (0.775–1.145)

Abbreviations: Q1-L, Lowest-Q1; IQR, interquartile range; Q3-H, Q3-Highest; SFA, saturated fatty acids; MFA, monounsaturated fatty acids; PFA, polyunsaturated fatty acids; HR, hazard ratio; CI, confidence interval. * *p* < 0.05; ** *p* < 0.01.

**Table 5:** Impact of dietary consumption on survival in patients with and without stroke.

	Stroke Q3-H (*n* = 232)		Non-Stroke Q1-L (*n* = 464)		Adjusted HR (95% CI)
	Dead (%)	Follow-Up	Dead (%)	Follow-Up	
Calories	57 (24.6)	56.1 ± 35.3	133 (28.7)	82.0 ± 32.5	0.622 ** (0.445–0.869)
Protein	54 (23.3)	55.9 ± 35.3	121 (26.3)	83.3 ± 32.0	0.491 ** (0.345–0.698)
Total fat	49 (21.0)	60.2 ± 34.7	121 (26.1)	81.3 ± 32.2	0.739 (0.522–1.046)
Total SFA	54 (22.9)	56.5 ± 33.7	120 (25.8)	83.7 ± 31.9	0.643 ** (0.459–0.902)
Total MFA	51 (22.0)	59.9 ± 34.1	123 (26.5)	81.8 ± 32.3	0.672 * (0.476–0.949)
Total PFA	58 (25.0)	57.6 ± 34.8	114 (24.6)	84.6 ± 32.9	0.620 ** (0.448–0.858)
Vitamin E	52 (22.4)	54.2 ± 34.3	119 (25.6)	84.7 ± 32.5	0.658 * (0.471–0.918)
Phosphorus	57 (24.6)	54.9 ± 34.9	120 (25.8)	83.6 ± 32.6	0.563 ** (0.403–0.787)
Magnesium	64 (27.6)	54.4 ± 34.0	125 (26.9)	85.0 ± 32.8	0.576 ** (0.419–0.791)
Sodium	65 (28.0)	56.3 ± 35.2	110 (23.7)	84.1 ± 32.4	0.476 ** (0.345–0.658)
Selenium	60 (25.9)	54.3 ± 35.0	125 (26.9)	82.4 ± 32.5	0.533 ** (0.382–0.743)
Caffeine	57 (24.6)	56.4 ± 32.0	114 (24.3)	83.3 ± 32.8	0.568 ** (0.409–0.791)
Moisture	42 (18.1)	46.9 ± 27.5	145 (31.3)	89.6 ± 33.7	0.397 (0.273–0.576)

Abbreviations: Q1-L, Lowest-Q1; Q3-H, Q3-Highest; SFA, saturated fatty acids; MFA, monounsaturated fatty acids; PFA, polyunsaturated fatty acids; HR, hazard ratio. * *p* < 0.05; ** *p* < 0.01.

## Data Availability

The datasets used in the current study are available on the NHANES website: https://www.cdc.gov/nchs/nhanes/ (accessed on 3 September 2022).
